# Kinematic primitives for walking and trotting gaits of a quadruped robot with compliant legs

**DOI:** 10.3389/fncom.2014.00027

**Published:** 2014-03-07

**Authors:** Alexander T. Spröwitz, Mostafa Ajallooeian, Alexandre Tuleu, Auke Jan Ijspeert

**Affiliations:** Biorobotics Laboratory, École Polytechnique Fédérale de LausanneLausanne, Switzerland

**Keywords:** motion primitives, locomotion patterns, central pattern generator, quadruped robot, passive leg compliance, entrainment, principal component analysis, walk and trot

## Abstract

In this work we research the role of body dynamics in the complexity of kinematic patterns in a quadruped robot with compliant legs. Two gait patterns, lateral sequence walk and trot, along with leg length control patterns of different complexity were implemented in a modular, feed-forward locomotion controller. The controller was tested on a small, quadruped robot with compliant, segmented leg design, and led to self-stable and self-stabilizing robot locomotion. *In-air* stepping and *on-ground* locomotion leg kinematics were recorded, and the number and shapes of motion primitives accounting for 95% of the variance of kinematic leg data were extracted. This revealed that kinematic patterns resulting from feed-forward control had a lower complexity (*in-air* stepping, 2–3 primitives) than kinematic patterns from *on-ground* locomotion (νm4 primitives), although both experiments applied identical motor patterns. The complexity of *on-ground* kinematic patterns had increased, through ground contact and mechanical entrainment. The complexity of observed kinematic *on-ground* data matches those reported from level-ground locomotion data of legged animals. Results indicate that a very low complexity of modular, rhythmic, feed-forward motor control is sufficient for level-ground locomotion in combination with passive compliant legged hardware.

## 1. Introduction

The overlapping fields of functional leg anatomy, leg and body compliance, and neuro-control in legged locomotion are intensively researched. Results potentially allow insights into the structure and functionality of the nervous system of animals. Not surprisingly, roboticists have started researching bio-inspired, legged robot systems, both on the functional morphological level, and the controller level. Though intrinsically limited (Webb, 2001), robots are beginning to be used as proof-of-concept platforms (Raibert et al., 1984; Raibert, 1990; Full and Koditschek, 1999; Ijspeert et al., 2007; Umedachi et al., 2010; Zhou and Bi, 2012).

In this experimental work we present results by comparing basic patterns measured from kinematic leg data from *in-air* stepping movements of a suspended legged, compliant robot, and from *on-ground* locomotion of the same robot during lateral sequence walk and trot. We applied as measure the number of significant principal components (PCs) extracted from joint-angle data of the robot's compliant, multi-segment legs. We compared four parameter setups, altering the robot's gait control parameters between walk and trot, its speed, and the modules and complexity of its locomotor drive signals. For the robot hardware, special attention has been paid to the in-series and in-parallel leg compliance. The robot was designed such that its leg's compliance and cable-driven actuation were the medium of *change of kinematic complexity* between feed-forward-sent and observed kinematic joint patterns, through emerging mechanical entrainment during level-ground (flat ground) locomotion. During *in-air* leg movement, leg-joints were not exposed to gravitational or inertial forces acting on the robot body (they were only exposed to those acting on the leg's own light-weight segments). The robot's legs were hanging freely *in-air*, and replayed motion patterns represented the kinematic complexity of the feed-forward locomotor controller. During *on-ground* locomotion, leg-joints were compressed by gravitational forces acting on the robot body, and Newtonian dynamics acting at the robot's body eventually deflecting the robot's compliant limbs. Self-stable and self-stabilizing locomotion only emerged if appropriate feed-forward patterns were sent to the robot. In all other cases, the robot would stumble, fall, or move only very poorly, i.e., very slowly or even backwards.

Physical, biological leg compliance was found to function as energy recoil mechanism, allowing animals to re-use negative work, and reduce metabolic cost of locomotion (Alexander, 1984, 1990, 1991; Biewener and Blickhan, 1988). Sources of compliance were found both in muscles and muscle complexes (Witte et al., 1994; Labeit and Kolmerer, 1995; Wilson et al., 2003), and in tendons and aponeuroses (Alexander, 1977; Witte et al., 1997; Biewener, 1998; Gregersen et al., 1998; Lichtwark et al., 2007). If biological systems rely that strongly on in-series and in-parallel compliant “locomotion hardware,” the nervous system producing motor control patterns has to be able to cope with leg compliance, and is required to send the corresponding control signals. Ivanenko et al. (2002) present experiments with walking humans, and *in-air* stepping with varying amounts of gravity, leading to no or limited sensory feedback through foot contact. They observe that during air-stepping “… motor patterns are transformed in simple harmonic angular motion of the lower limb segments associated with alternating activation of antagonist muscles” (cf. Ivanenko et al., 2002, p. 3087). Although they conclude on the role of peripheral sensory input, we are building in this work on the idea that the complexity of leg kinematics during locomotion can be increased through compliant, and purely mechanical components of the locomotor apparatus and its interactions with the ground. In turn, this could mean that the underlying layers of motor control do not need to send as complex control signals as one might have guessed from kinematic studies, while still achieving a sufficiently complex, adaptive kinematic output.

The spring-loaded inverted pendulum (SLIP) framework describes how an abstracted, energy conservative point-mass system can make use of a compliant, in-series elastic leg design to self-stably run (Blickhan, 1989; Seyfarth et al., 2002). In a SLIP simulation, minimal control effort is required to stabilize locomotion. Control of the leg's angle of attack is necessary, together with monitoring the system's hip apex height. A feed-forward SLIP controller will lead to self-stable locomotion patterns, also under the influence of small perturbations. Full and Koditschek (1999) explain the SLIP model as a “template.” A biological or robotic legged system still requires an “anchor,” to map the system mechanics to the template.

In this work, central pattern generator (CPG) control signals in feed-forward mode were implemented as high-gain position signals from the robot's RC-servo motors, actuating hip joints and leg length. Interpreting CPG output patterns as position signals presents an abstraction and simplification of animal motor control and actuation. In animals, motor control signals of the nervous system are interpreted by sets of antagonistic muscles and muscle groups to produce joint torques (Inman et al., 1952). Hence, animals can control their limbs in many modes, including “position-control” (muscle lengths leading to joint angles), but also with adjustable joint torques (Winter, 1983; Fischer and Blickhan, 2006).

With the help of a robotic tool like Cheetah-cub robot, this work's intention is to shed light on the interplay between rhythmic, modular feed-forward motor control, and the mechanical entrainment leading to stable gait patterns. Mechanical entrainment was a result from an in-series and in-parallel segmented, robotic, bio-inspired leg design, appropriate actuator control patterns, gravity and body dynamics, and ground contact during locomotion. Though not within the topic of quadruped legged walk and trot locomotion, entrainment in articulated robotic systems has been looked upon earlier; Lungarella and Berthouze (2002) designed a setup with a swinging humanoid robot, showing that physical entrainment led to a larger basin of attraction for the space of control parameters leading to stable swinging motions. Entrainment was also achieved at the presence of non-linear mechanical coupling of the humanoid to its environment (Berthouze and Lungarella, 2004). This is interesting because ground contact of Cheetah-cub robot also presents a non-linear perturbation (alternating leg swing and stance phase).

Here, the simplified case of a position-controlled system with serial compliance allows us to focus on few components, and dismiss the effects of additional control and hardware complexity (e.g., explicit control feedback loops, corresponding controller architecture, or torque control strategies). As mentioned earlier, animals feature components of (simple) in-series leg compliance (Alexander, 1984; Gregersen et al., 1998). This morphological feature works also well for robots. In-series compliance can lead to reduced impact forces, what consequently can reduce the control complexity (Meyer et al., 2006).

In the presented experiments, change of complexity between the directly commanded *in-air* leg kinematics and the *on-ground* locomotion leg kinematics emerged through the robot's compliant leg design and the interactions with the ground. At level-ground locomotion, periodic leg length shortening is caused (a) by signals of the robot's feed-forward controller and (b) by the robot-body's pitch, roll, vertical, and translational body movements (Figures 2-2–5). The inertia- and gravity-induced robot body and leg length movements present a major difference between Cheetah-cub robot and the controller applied in this work, and other feedback-controlled and body-stabilized quadruped robots (Raibert et al., 1986; Havoutis et al., 2013). If one would design a similar set of experiments, but sent feed-forward signals to a high-gain position controlled robot with stiff leg design, its kinematic data from *in-air* running would (largely) match that of *on-ground* running experiments. Hardware springs and cable clutches are not the only way to achieve deviating, adaptive joint kinematics. Alternative setups can facilitate low-gain position controlled actuators, or more generally torque or force controllers (Buchli et al., 2009; Valenzuela and Kim, 2012). These setups, however, require explicit feedback control.

Kinematic leg patterns of Cheetah-cub robot were extracted by principal component analysis (PCA; Krzanowski, 1988; Jolliffe, 2002) on the normalized kinematic leg data, for both *in-air* leg movements, and *on-ground* locomotion. Applying PCA on kinematic leg data from recording of locomotion experiments, or corresponding electromyographic data of the participating muscles has become a common tool in biology, and neurobiology. Dominici et al. (2011) present in a comparative study basic patterns derived from electromyographic (EMG) data of stepping human neonates, toddlers, pre-schoolers, and adults. The authors compare these results to data of neonatal rats, and adult quadruped animals such as cats and monkeys. They report that human neonate stepping and neonatal rat stepping can be represented by two basic patterns, and that human toddler locomotion activation patterns along with all adult quadruped animals share a similar pool of four basic patterns. However, toddler and human adult patterns show differences: “… the four patterns [of human adults] were accurately timed around the four critical events of the gait cycle …” (cf. Dominici et al., 2011, p. 998). Basic patterns from toddler locomotion were less time structured. Dominici et al. (2011) conclude that the increase in patterns is caused by continuous learning, until adulthood. The similarity between toddler data, and basic patterns of adult quadrupedal animal locomotion provides a possible explanation as to where gait patterns in vertebrates originate: central pattern generators located in the spinal cord (Delcomyn, 1980; Grillner, 1985; Ijspeert, 2008). Moro et al. (2013a) extracted four basic patterns (kinematic motion primitives, kMPs) from horses, for the three gaits walk, trot, and gallop (kMPs account for 93% and 97% of the kinematic data). Moro et al. (2013b) found five kMPs accounting for almost all variance of human walking and running kinematics. Ivanenko et al. (2004) reported five basic muscle activation patterns accounting for almost all variance of muscle activation, during human walking. Koditschek et al. (2004) reported observations on retrieving basic patterns from running cockroaches. Despite the animal's very high number of degrees of freedom—a cockroach is six legged and has multi-segment legs—“… a single component represen[ted] over 80% of the variation …” (cf. Koditschek et al., 2004, p. 256), for very fast running. The authors report that almost all variations were captured by three basic components, leading to the conclusion that a very simple neural controller was likely responsible for the motor control of this insect. The above findings from insects and mammals suggest that basic locomotion on level-ground requires three to five basic patterns, and possibly fewer for very fast locomotion. It is intriguing to be able to hypothesize and through robot hardware implementation and experimentation, test the interplay between locomotion controller, robot morphology, and locomotion patterns. Although direct conclusions can only be drawn for the artificial, robotic system, similar designs of both systems and similar results for the task of locomotion can provide insights into animal locomotion control, and how neuro-control is interacting with bio-mechanical components.

**Figure 1:**
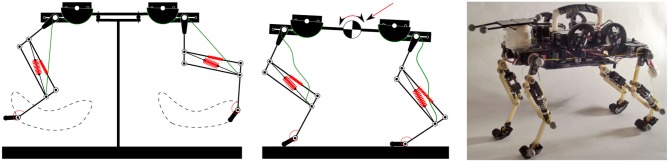
**Schematic presentation of the *in-air* experiment (left), the *on-ground* experiment (middle), and a picture of the real robot (right).** For the *in-air* experiment, feed-forward locomotion patterns (permutations of trot, lateral sequence walk, locomotion frequencies of 2.5 and 3.5 Hz, two different control pattern types) were sent to the robot, while the robot's body was mounted on a stand, with its legs swinging freely *in-air*. At the *on-ground* experiment, the quadruped robot walked and trotted freely on level-ground, with an average speed between 0.45 and 0.9ms^−1^. Identical motor control patterns were sent to both *in-air* and *on-ground* experiments, for each experiment type. In all experiments, resulting kinematic patterns (leg angle and leg length) were recorded. Change of complexity of kinematic primitives between *in-air* and *on-ground* derived from ground contact, and the robot's compliant leg design. Further details of Cheetah-cub robot's leg design and compliance are available in Figure 2.

We recorded joint-angle leg kinematics of our quadruped robot, for two situations: *in-air* trotting movements, versus robot locomotion *on-ground* (Figure 1). With its legs swinging *in-air* and without contacting the floor, the kinematics of the robot's low-inertia leg-joints followed the commanded patterns. Once Cheetah-cub robot is placed *on-ground* to walk or trot, the interplay between ground contact, in-series leg compliance and spring deflection, and body movements alters the complexity of its leg kinematics. In all experiments documented, motor control patterns where sent feed-forward. Hence, the observed changes between *in-air* and *on-ground* leg kinematics were caused by ground contact and mechanical entrainment.

The experiments of this work are intended to inform on the potential interplay of a compliant, legged system, such as found in legged animals or robots, and its motor control. In animals, the interplay of the brain, spinal cord, peripheral nervous system (PNS), morphology, and intrinsic, mechanical properties (springiness, damping, inertial moments, lengths, speed and force properties) plays a key role for its locomotion capabilities. Studies like Ivanenko et al. (2002) or Hägglund et al. (2013) show that understanding the role, structure, and interplay of locomotor components in animals is difficult. With a largely simplified robot “hardware model” we can focus on the interplay of only a few components. Specifically, this robot features only these locomotor components: a feed-forward, oscillatory motor controller (we implemented a central pattern generator, but any other controller with similar features could be applied), and the mechanically compliant, bio-inspired leg structure. Cheetah-cub robot is stripped off any task-level control feedback, the motor control CPG is purely running in feed-forward mode. Self-stable and self-stabilizing locomotion was a product of appropriate motor control patterns (derived through systematic testing, see (Spröwitz et al., 2013), the robot's compliant leg design, and the mechanical entrainment of these components, through ground contact during *on-ground* locomotion.

Establishing a reduced experimental setup, without any task-level feedback from the nervous system, is hard to achieve in live, locomoting animals. Possibilities for modulation of PNS pathways include preparations with drugs, lesions (see for example Grillner and Zangger, 1979), or more recently introduced methods like light-evoked activation and deactivation of spinal cord and PNS components (Daou et al., 2013; Hägglund et al., 2013). The vast number of publications in the field shows the complexity of locomotion generation and control in animals. From this perspective, the use of a legged robot with a programmable motor controller and dedicated hardware presents a diametrical, bottom-up approach to analyze the interplay of only its featured, however, much simpler locomotor components.

As our **first hypothesis** (H1-1) we expect four to five basic patterns accounting for 95% of the variance of kinematic leg data, at low-speed and mid-speed robot locomotion, as observed from legged animals (Ivanenko et al., 2004; Dominici et al., 2011; Moro et al., 2013a,b). Further, Koditschek et al. (2004) had reported a decreasing number of motion primitives at high locomotion speed, for a simpler biological legged systems with leg compliance. Hence, we expect a similar trend: a decreasing number of *on-ground* PCs with increasing robot speed (H1-2).

In addition, we looked at the number of *in-air* stepping PCs, versus the number of *on-ground* locomotion PCs. The interaction of ground contact, feed-forward controlled compliant legs, and the naturally emerging pitch and roll body movements produced a self-stable walk or trot gait (Spröwitz et al., 2013). This richer, additional pattern through mechanical entrainment should be visible as a higher number of observed basic principal components for *on-ground* locomotion, compared to non-contact, *in-air* stepping. *In-air* stepping presents virtual locomotion patterns with rigid, non-compliant legs. No ground contact deflects the serial leg springs out of their slack length, and recorded complexity of *in-air* kinematic data represents effectively the complexity of Cheetah-cub's feed-forward controller. As our **second hypothesis** we expect a higher number of basic patterns for *on-ground* locomotion, compared to *in-air* stepping (H2).

This paper is organized as follows: in section 2 we give a short overview of the robot's locomotion controller and hardware. We provide details of data recording and processing, and of the extraction of basic primitives from the recorded kinematic data. In section 3 we present the results from the four proposed experiments for walk and trot locomotion, *in-air* and *on-ground*. In section 4 we discuss results and their implications for biological and robotic systems. Finally, we conclude the paper.

## 2. Materials and methods

The first part of this section covers a brief description of the robot's hardware. For a more throughout description please refer to (Spröwitz et al., 2013). Next, this section provides information about the experimental tools and setup, and details about the applied principal component analysis. Cheetah-cub robot's gait controller based on a central pattern generator (CPG in feed-forward mode) is explained. Videos of Cheetah-cub robot running can be found at http://biorob2.epfl.ch/utils/movieplayer.php?id=209 and http://biorob2.epfl.ch/utils/movieplayer.php?id=207.

### 2.1. Quadruped robot hardware

**Figure 2:**
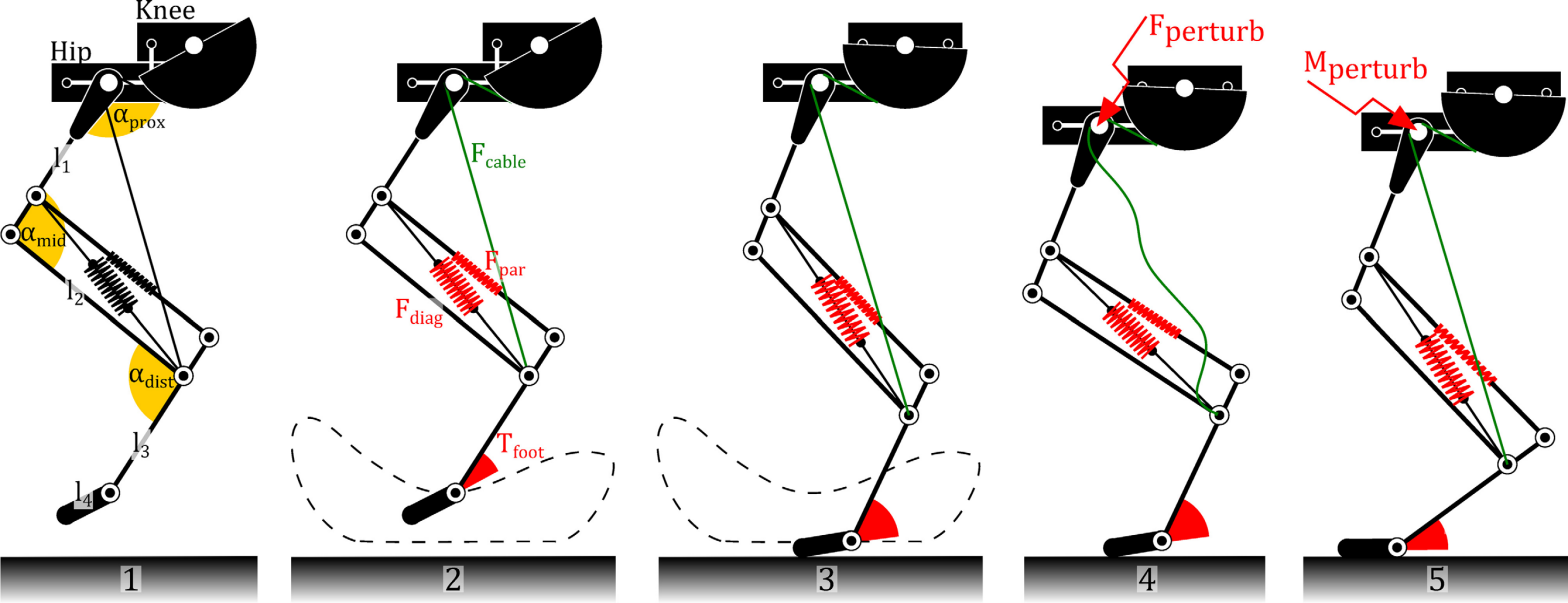
**Cheetah-cub leg mechanism, and leg compliance.** A single leg is shown abstracted, detailed leg segment ratios are omitted for clarity, robot heading direction is to the left. **(1)** shows the three leg angles α_prox_, α_mid_, and α_dist_. Hip and knee RC servo motors are mounted proximally, the leg length actuation is transmitted by a cable mechanism. The pantograph structure was inspired by the work of Witte et al. (2003) and Fischer and Blickhan (2006). **(2)** The foot segment describes a simplified foot-locus, showing the leg in mid-swing. For ground clearance, the knee motor shortens the leg by pulling on the cable mechanism (green, *F*_cable_). *F*_diag_ is the major, diagonal leg spring. Its force extends the pantograph leg, against gravitational and dynamic forces. **(3)** The leg during mid-stance. **(4)** In case of an external translational perturbation, the leg will be compressed passively. **(5)** If an external perturbation torque applies e.g., through body pitching, the leg linkage will transmit it into a deflection of the parallel spring, not of the diagonal spring.

**Figure 3:**
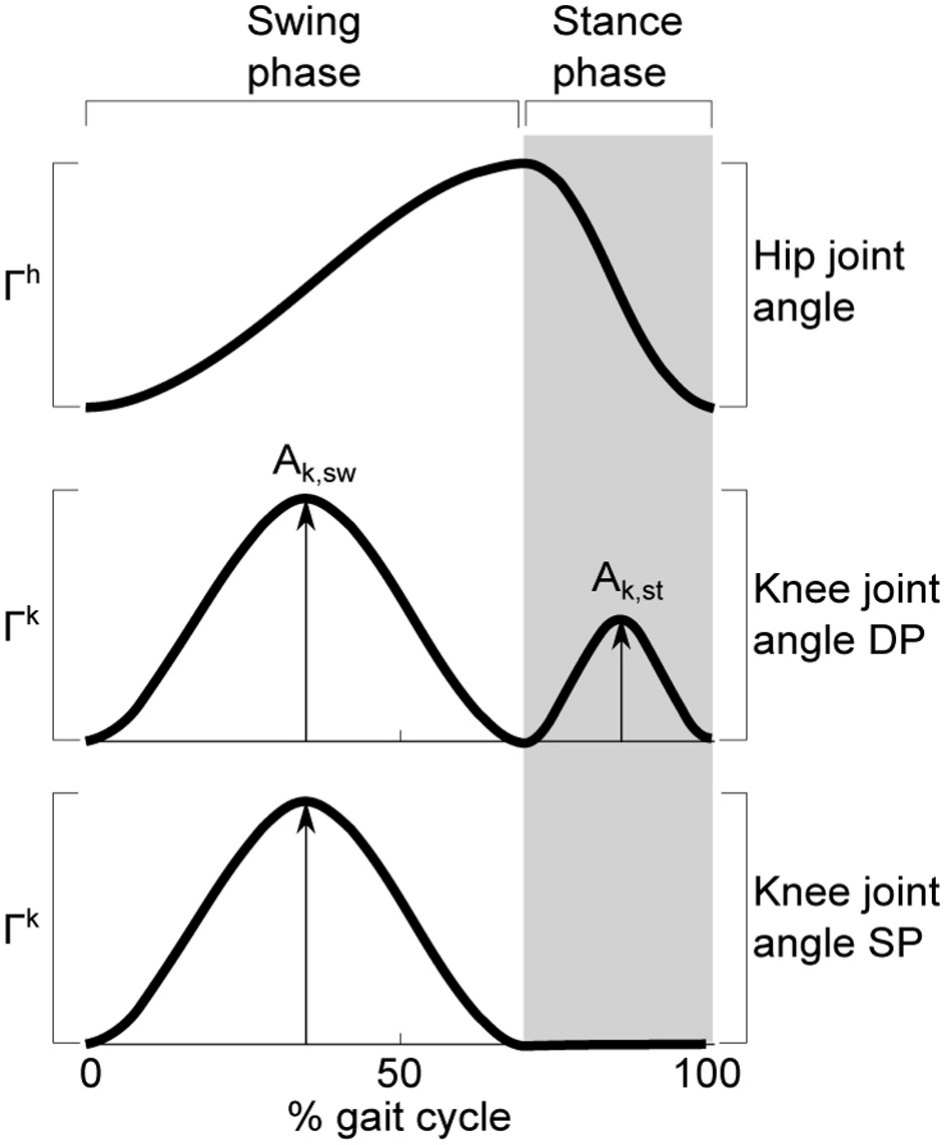
**Schematic presentation of the control signals for the two servo motors per robot leg: hip joint-angles (top, Γ_**h**_) and knee joint-angle (center, bottom, Γ_**k**_) for one stride cycle.** Swing phase is plotted on the left side (transparent background), stance phase on the right side (gray background). The *double-peak* (DP) knee signal activates the knee motor twice per stride cycle. The leg is flexed stronger during swing phase (larger amplitude), and less flexed during stance phase (***A***_**k,st**_). The *single-peak* (SP) knee motor activation signal triggers only during swing phase (***A*_k,sw_**). Leg flexing in SP mode during stance phase emerges through inertia and gravity acting on the robot body, compressing the compliant stance leg at ground contact. For DP knee activation, active actuator leg shortening overlapped with inertia-induced leg shortening (Spröwitz et al., 2013).

**Figure 4:**
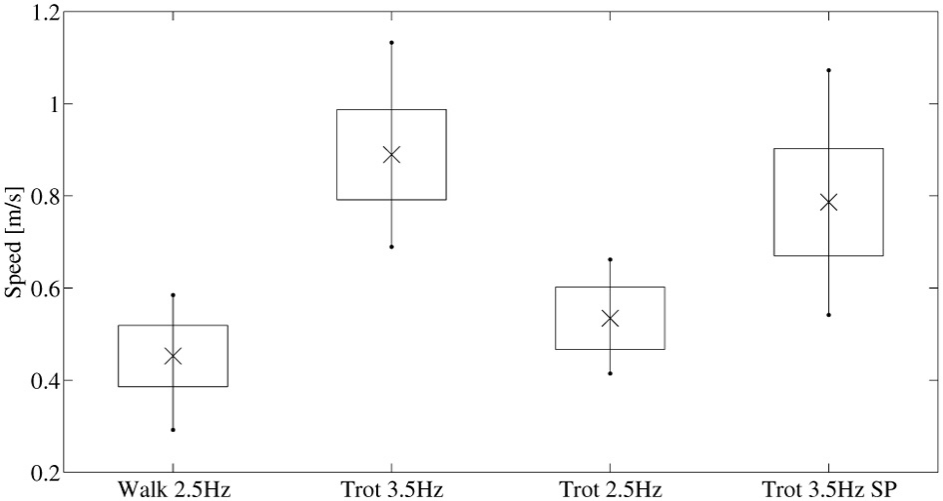
**Average speed values of the four experiments, sorted by gait type (walk, trot) and gait frequency (*f* = 2.5, 3.5 Hz).** The 3.5 Hz trot applied a single-peak knee-trajectory (SP), the three remaining experiments applied double-peak knee-trajectories (DP). For the applied RC servo motor voltage of 12 V, a trot gait speed of 0.9ms^−1^ (FR = 1) is about the maximum average speed.

Cheetah-cub robot's leg design is based on a mammalian, animal-inspired pantograph mechanism (Witte et al., 2003; Fischer and Blickhan, 2006). An automatic, cable-based clutch mechanism, proximal actuation, and a compliant foot joint enhanced the original, bio-inspired hardware blueprint (Spröwitz et al., 2013). Each robot-leg was individually controlled by two RC servo motors. The leg length (knee) actuator actively *flexed* the leg via a cable mechanism, antagonistic to the diagonal leg spring (Figure 2-2). The cable mechanism also works as an automatic decoupling mechanism. It goes slack if external forces are applied to the leg (Figure 2-4). The robot's proximal actuator was directly mounted between body and leg. It protracted and retracted front and hind legs.

The robot's body was implemented as a stiff plate, only legs provided compliance. Three leg springs are acting in this leg design, under different load conditions (Figure 2): *F*_diag_ is the in-parallel spring to the cable actuation, and provides anti-gravity support. *F*_par_ is the spring replacing one of the struts of the linkage mechanism. Under tension it provides an in-series leg elasticity. The third spring is located in the most distal leg joint. It is a helical spring and provides serial foot torque. In sum, this presents a very compliant leg design with a very low leg stiffness, in comparison with biological systems. The linearized vertical leg stiffness of two in-parallel Cheetah-cub robot legs is about 0.25 kN/m, for a static measurement with isolated legs. During fast trotting locomotion (Froude number speed of FR = 1.0), a leg stiffness of *F*_vert_/Δ*l* = 0.65 kN/m was recorded. Leg stiffness for *running*, quadruped animals of this body weight, but at faster speed, are documented to be almost twice as high (*k*_leg_ = *M*^0.67^ = 1.05 kN/m, using the convention of treating all ground-contacting legs as one, (Farley et al., 1993). Compared to a young cat of equal weight, Cheetah-cub robot exhibits a more crouched leg posture. This is generally associated with a lower overall leg stiffness through bigger effective lever arms.

In all experiments, the robot was tethered to a power supply through a long, light-weight power cable. CPG computation, RC servo motor control signal generation, and wireless communication were controlled from a single board computer, mounted on the robot's body.

### 2.2. Dataset, extraction, and experimental setup

The robot was controlled with four different sets of control parameters (Figure 4). The robot ran at two different gait patterns (lateral sequence walk, and trot), a speed range from mid-speed to higher-speed, and two different knee-control strategies. Thus, the robot exploited its body dynamics for different gait patterns, control complexities, and dynamical speed conditions: (a) Lateral sequence walk gait with a locomotion cycle frequency of 2.5 Hz, with double-peak knee deflection (DP, Figure 3), at medium robot speed. (b) Trot gait with a locomotion cycle frequency of 3.5 Hz, with DP knee actuation, at high robot speed. (c) Trot gait with a locomotion cycle frequency of 2.5 Hz, with DP knee actuation, at medium robot speed. (d) Trot gait with a locomotion cycle frequency of 3.5 Hz, with a single-peak knee actuator signal (SP), at higher [compared to (a) and (c)] robot speed. Single-peak and double-peak leg length control signals, and hip-joint control signals are plotted for one locomotion cycle in Figure 3. All robot runs were repeated 10 times, and between 30 and 60 stride cycles were extracted for each gait. Kinematic robot data was recorded with a motion capture (MOCAP) system, based on infrared reflective markers of 11 mm diameter. Twelve MOCAP cameras (Optitrack s250e, Naturalpoint, Inc., 2011) were mounted at 1.20 m and 2.30 m height, positioned in a large rectangular arena around the locomoting quadruped robot. Cameras observed a volume of 1.5 m width, 4 m length, and 0.5 m height. MOCAP data were captured at *f* = 250 fps. Marker trajectories were processed and cleaned with Arena software (Naturalpoint, Inc., 2011). Unlabeled markers were labeled in *Mokka* (Barré and Armand, 2014). Data was loaded in Matlab (MATLAB, 2009, v. 7.9) with *b-tk* framework (Barré and Armand, 2014). All marker trajectories were low-pass filtered, with an 18 Hz cut-off frequency.

For *in-air* experiments, Cheetah-cub was mounted on a small stand in the center of the arena. Its legs were hanging freely in the air, and MOCAP cameras recorded leg kinematics. For *on-ground* experiments, Cheetah-cub ran the full length of the motion capture arena distance (4 m), without restraints. The robot was powered externally by a power tether. Cheetah-cub's design includes no explicit degree of freedom for changing direction, i.e., adduction or abduction. Before reaching its steady state and before recording was started, the power tether was, in a few cases, used to correct the robot's heading. During recording, the robot would walk or trot while the tether was carefully kept loose. The robot was started from the ground, data was recorded once it reached steady state. This happened typically after less than three locomotion cycles. Markers were attached on the robot's right side of fore and hind limb, on the proximal leg joints, mid-leg joints, and feet (Figures 1, 2). Using leg kinematics, angles of proximal (α_prox_), middle (α_mid_), and distal (α_dist_) joints were calculated (Figure 2-1). As the robot leg's parallel spring and foot spring work in-series, deflection of the distal leg segment and the foot segment were combined for simplification into a single angle (α_distal_). Recorded locomotion stride cycles were synchronized based on *pi*/2 crossing of hip angle of the virtual leg (hip to foot), at mid-swing. The end of each stride cycle was calculated from the inverse stride frequency. Finally, all cycles were divided into 100 samples per cycle, using Piecewise Cubic Hermite Interpolating Polynomial (Fritsch and Carlson, 1980).

### 2.3. Principal component analysis, PCA

Kinematic primitives describing the main components of the kinematic leg dataset can be computed by matrix factorization (Strang, 2003). We applied Principal Component Analysis (Krzanowski, 1988; Jolliffe, 2002) to implement matrix factorization (similar to Fod et al., 2002; Bizzi et al., 2008). The obtained data are represented with **X***_n_o_, n_v__*, with *n_o_* being the number of observations, i.e., the number of samples per cycle, and *n_v_* being the number of variables, i.e., the number of cycles. The dataset was first normalized $\tilde{X}$*_n_o_, n_v__*, so each cycle had a zero mean and a standard deviation equal to one. The covariance matrix of the normalized dataset was then calculated, obtaining **Σ***_n_v_, n_v__*:

(1) $$\Sigma =\frac{1}{N-1}\sum_{i\;=\;1}^{N}{\left({\tilde{X}}_{i}-\bar{X}\right)}^{T}({\tilde{X}}_{i}-\bar{X})$$

where ${\tilde{X}}_{i}$ is the *i*-th observation in $\tilde{X}$, and *X* is the mean observation. Principal components of the covariance matrix were extracted, obtaining the loading vectors **v**_*i*_, *i* = 1..min(*n_o_* − 1, *n_v_*), and the respective eigenvalues λ_*i*_, sorted in descending order. Typically a low number of principal components are sufficient to account for a big part of variance. If the first *n_s_* components account for a percentage variance (e.g., *n_s_* components account for 95% of the variance, for all results in this work), then *n_s_* primitives are obtained by projecting the normalized dataset on the most significant loading vectors **v**_*i*_, *i* = 1..*n_s_*:

(2) $$p_{i}=\tilde{X}v_{i}$$

### 2.4. Locomotion control with central pattern generator

Central pattern generators (CPG) were successfully applied to generate locomotion patterns for legged and other robots (Fukuoka et al., 2003; Ijspeert, 2008; Spröwitz et al., 2008, 2013; Sato et al., 2011). We applied a CPG implemented as a network of coupled oscillators to rapidly and conveniently encode a feed-forward control signal with explicit, legged locomotion-relevant input parameters such as duty factor, hip amplitude, leg length, and locomotion frequency. Cheetah-cub is a RC-servo motor controlled quadruped robot. The CPG provides for smooth trajectory transition at gait initialization, because of damping terms in the CPG equations. The CPG controller was running feed-forward, i.e., it was streaming a position signal to the RC-servo motors, without incorporating external feedback. Cheetah-cub robot's CPG controller consisted of two modules, a hip controller, commanding the hip motor, and a knee controller, commanding the leg length through a proximally mounted knee motor. Two knee control strategies (single-peak SP, and double-peak DP) were implemented. An example locomotion cycle is provided in Figure 3. Top and center plots show a gait applying double peak knee-signals (DP). Top and bottom plots show a gait with a single-peak (SP) knee-signal. The hip signal (top plot) is identical for SP and DP gaits. We used previously derived CPG parameters for trot gait (Spröwitz et al., 2013). The hip-joint-driving CPG consisted of a network of four phase-coupled oscillators, each oscillator controlled one hip joint. The gait was switched from lateral sequence walk (cf. Hildebrand, 1989) to trot by setting the phase shift between hip oscillators accordingly (Righetti and Ijspeert, 2008). Knee oscillators of the corresponding knee were coupled serially to their hip oscillator. The range of speeds obtained for walk and trot is shown in Figure 4.

## 3. Results

This section provides the results from four experiments, each for *in-air* stepping and *on-ground* locomotion of the robot. The number and shapes of basic components accounting for at least 95% of the variance of the kinematic leg data are provided.

### 3.1. Double-peak (DP) knee pattern, walk, *f* = 2.5 Hz

**Figure 5:**
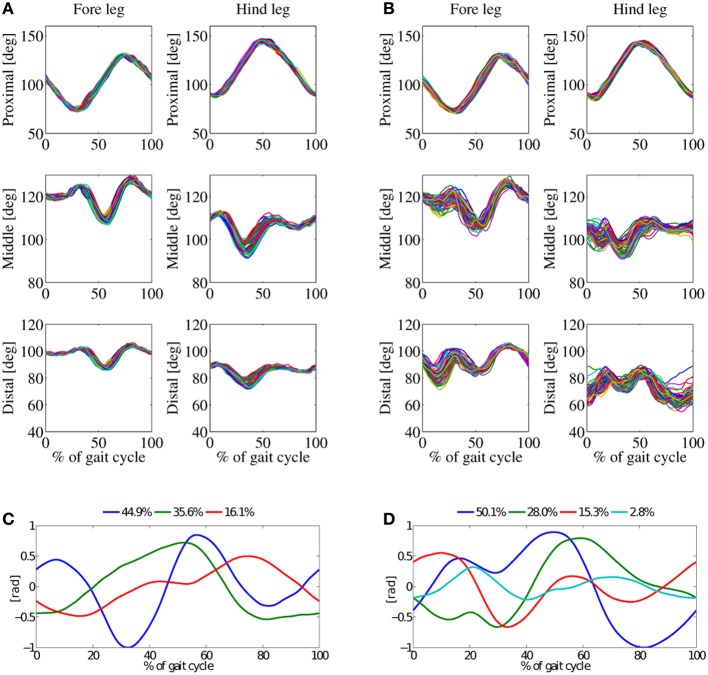
**Results from walk-gait experiments, at a locomotion frequency of 2.5 Hz, with double-peak knee activation patterns. (A)** Joint angles for *in-air* experiment, and **(B)** joint-angles for *on-ground* experiment. **(C)** Principal components (PC) for the *in-air* experiment, and **(D)** PCs for the *on-ground* experiment. Three basic patterns of the *in-air* experiment sum up to 97%, the four basic pattern of the *on-ground* experiment to 96%.

Figure 5 shows joint-angle data *in-air* and *on-ground* (Figures 5A,B), and the corresponding principal components (Figures 5C,D). Average robot speed for this experiment was 0.45ms^−1^, around 2.3 body lengths per second. Figures 5C,D indicate that more than 97% of the *in-air* patterns can be presented by three principal components, and 96% of the *on-ground* joint-angles kinematics by four principal components. The first PC of *in-air* and *on-ground* represents almost 50% of the kinematic data, for both cases (Figure 9A).

### 3.2. Double-peak knee pattern, trot, *f* = 3.5 Hz

**Figure 6:**
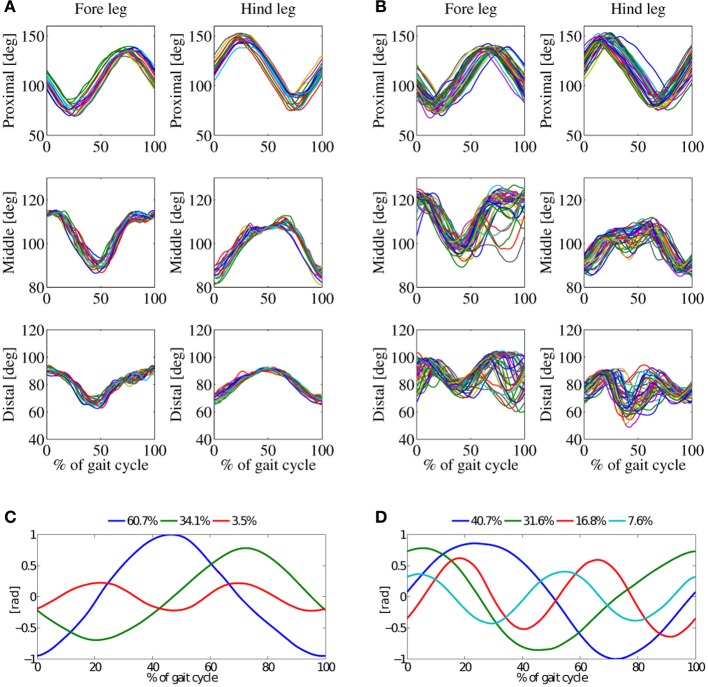
**Trot-gait experiments, locomotion frequency 3.5 Hz, average robot speed was 0.9ms^−1^, maximum speed was 1.1ms^−1^, double-peak knee activation patterns. (A)** Joint angles of *in-air*, and **(B)** joint-angles of *on-ground*. **(C)** Principal components for the *in-air* experiment, and **(D)** PCs for the *on-ground* experiment. Three basic patterns of the *in-air* experiment sum up to 98%, the four basic pattern of the *on-ground* experiment to 97%.

Average robot speed for the 3.5 Hz trot gait with double-peak knee actuation was the highest of all four experiment types (0.9ms^−1^). Higher average robot speed is possible with higher motor coil voltage, up to 1.42ms^−1^ (Fr = 1.3, Spröwitz et al., 2013). However, the power consumption becomes so large that motors would break at longer experimentation, caused by coil overheating. A speed of 0.9ms^−1^ was a compromise between fast robot locomotion, around 4.5 body lengths per second, and a repeatable, robust experimental setup. The results in Figure 6 show three basic patterns for *in-air* stepping (98%), and four basic patterns for *on-ground* locomotion (97%). Figure 9B shows that the first *in-air* PC of this gait accounted for more than 60% of the variance of the kinematic data.

### 3.3. Double-peak knee pattern, trot, *f* = 2.5 Hz

**Figure 7:**
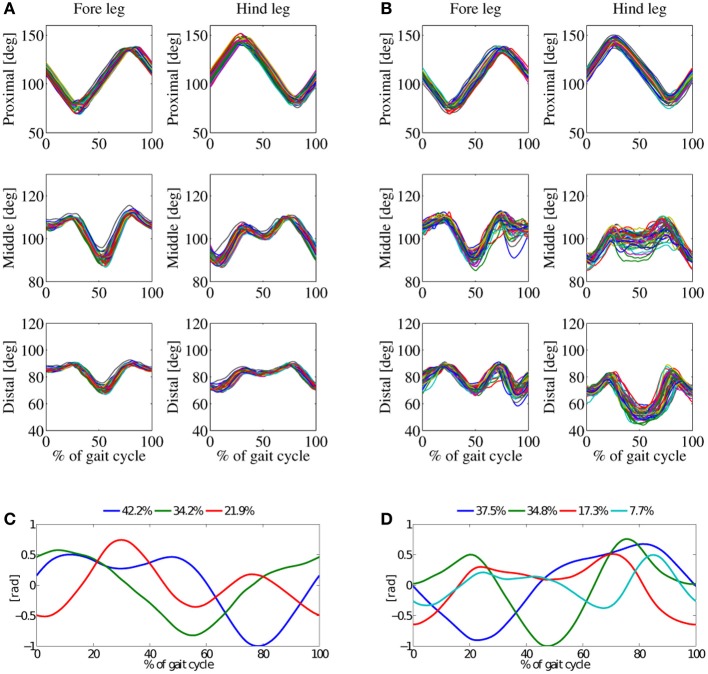
**Results from trot-gait experiments at a locomotion frequency 2.5 Hz.** Average robot speed was 0.55ms^−1^, double-peak knee activation patterns. **(A)** Joint angles for *in-air* experiment, and **(B)** joint-angles for *on-ground* experiment. **(C)** Principal components (PC) for the *in-air* experiment, and **(D)** PCs for the *on-ground* experiment. Three basic patterns of the *in-air* experiment sum up to 98%, the four basic pattern of the *on-ground* experiment to 98%.

In this experiment the robot trotted at mid-speed level, at *v*_*av*_ = 0.55ms^−1^. This was little more than the average speed of the robot for walk-gait experiment (0.45ms^−1^), at the same gait frequency. Three basic patterns account for 98% of the variance of the *in-air* stepping data (Figures 7, 9C). Four basic components account for 97% of the variance of the *on-ground* locomotion joint-angle data.

### 3.4. Single-peak (SP) knee pattern, trot gait, *f* = 3.5 Hz

**Figure 8:**
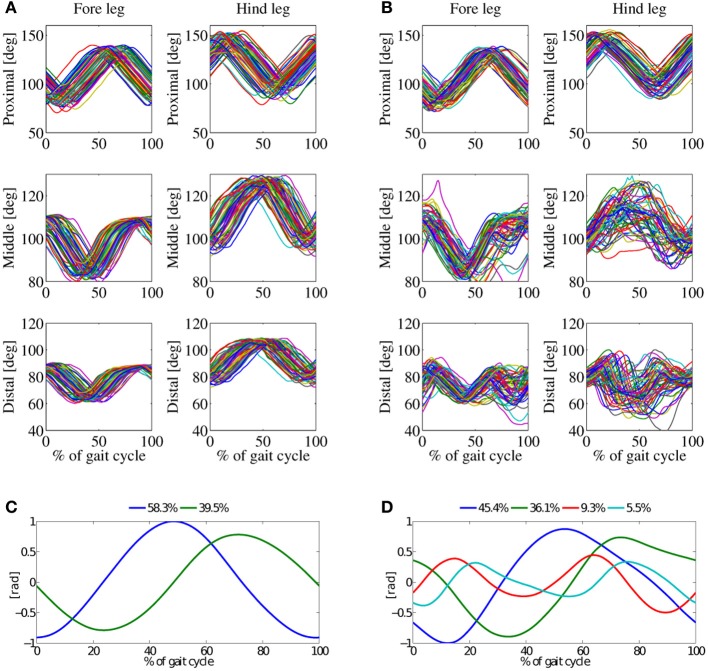
**Results from the trot-gait experiments, at a locomotion frequency 3.5 Hz.** The average robot speed was 0.8ms^−1^, single-peak knee activation patterns. **(A)** Joint angles for *in-air* experiment, and **(B)** joint-angles for *on-ground* experiment. **(C)** Principal components (PC) for the *in-air* experiment, and **(D)** PCs for the *on-ground* experiment. Two basic patterns of the *in-air* experiment sum up to 98%, the four basic pattern of the *on-ground* experiment to 96%.

**Figure 9:**
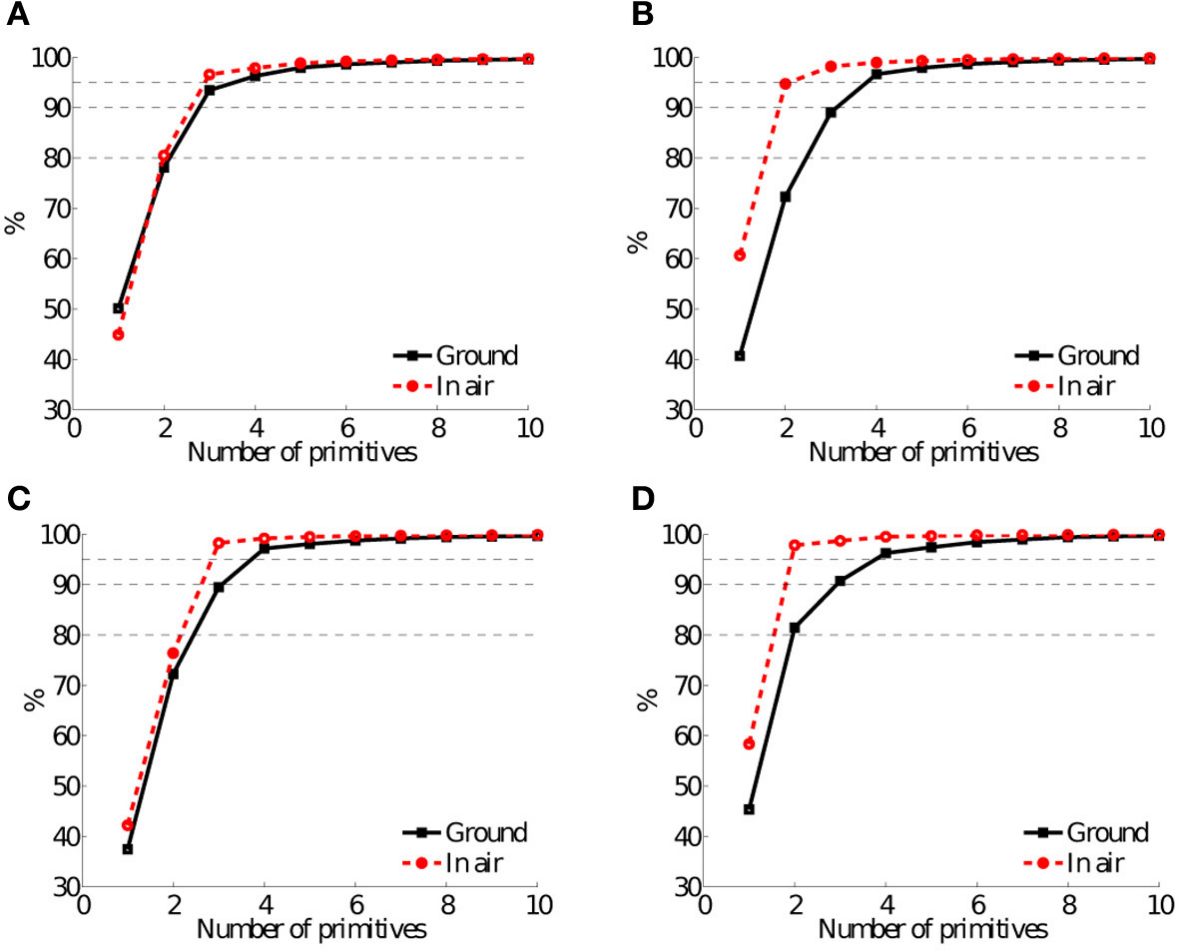
**The percent variance accounting for the first 10 primitives, as a function of the number of primitives, for *in-air* stepping (red, dashed lines) and *on-ground* (black, solid lines) locomotion patterns.** Horizontal, black, dashed lines indicate 80%, 90%, and 95% of the variance. In this article, we used a 95% of the variance interval (top, dashed, black, horizontal line). This results in between 2 and 4 primitives to account for ≥95% of the variance. The single peak (SP) trot gait at 3.5 Hz **(D)** showed the largest change from *in-air* stepping to *on-ground* locomotion: 2 primitives accounted for more than 95% of the variance of *in-air* stepping data, and 4 primitives accounted for the same variance threshold, for *on-ground* locomotion. In the three other **(A–C)** experimental setups 3 primitives accounted for at least 95% of the variance of kinematic *in-air* stepping data, and 4 primitives accounted for the same threshold of *on-ground* locomotion. **(A)** Walk, 2.5 Hz, DP. **(B)** Trot, 3.5 Hz, DP. **(C)** Trot, 2.5 Hz, DP. **(D)** Trot, 3.5 Hz, SP.

Single-peak (SP) knee actuation based locomotion was only stable above a speed of 0.55ms^−1^. The single activation burst of the knee actuator, effectively shortening leg length, is triggered during swing phase. This provides leg ground clearance to freely swing the leg and foot forward. Without leg length actuation during stance phase, leg shortening relies solely on inertia and gravity forces acting on the robot body compressing (flexing) the leg. This necessary amount of inertial energy explains the minimum required speed for SP experiments. Figure 8 shows two almost sine-shaped PCs for *in-air* stepping. Both PCs account for 98% of the variance of the kinematic joint-angle data, the first PC accounts for 58% of the variance, the second PC 40% of the variance. *On-ground* locomotion showed four PCs, accounting for 96% of the variance. The number of *in-air* PCs differed, compared to all other experiments (Figure 9D). The major change was a switch of the complexity of the knee-control signal, from double-peak to single-peak. This reduction was reflected in the *in-air* PC data, however, *not* in the *on-ground* PC data.

### 3.5. Combined data

**Figure 10:**
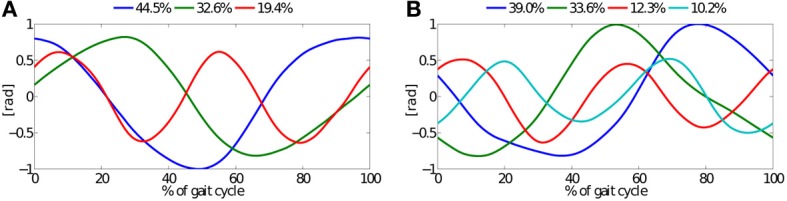
**PCs extracted from joint-angle trajectories of all four experiments, i.e., walk and trot, single (SP) and double-peak (DP) knee actuation, and 2.5 and 3.5 Hz locomotion frequency. (A)** PCs from *in-air* experiment, and **(B)** PCs from *on-ground* experiment. Three basic patterns account for 97% of the variance during *in-air* stepping, 95% for *on-ground* locomotion.

In Figure 10, PCs of *in-air* leg movements and *on-ground* locomotion of all four experiments are depicted. This common pool of all collected joint-angle kinematics includes lateral sequence walk and trot gait data. The corresponding joint-angle plot was omitted, it basically covers the entire plot area. Three *in-air* PCs account for 97% of the variance, and four *on-ground* PCs account for 95% of the variance.

## 4. Discussion

We presented results of locomotion patterns *in-air* and *on-ground*, for the two gait types walk and trot. The robot ran at average forward speeds between *v*_*av*_ = 0.45 ms^−1^, more than 2 body lengths per second, and *v*_*av*_ = 0.9 ms^−1^, around 4.5 body lengths per second. All *on-ground* locomotion experiments showed four principal components. *In-air* experiments revealed either two PCs (single-peak knee controller) or three PCs (double-peak knee controller), accounting for at least 95% of the variance of the kinematic leg data.

Stable walk and trot gait pattern were derived by encoding joint control patterns as a set of coupled oscillators (CPG), and manually tuning CPG parameters. For all speeds and gaits, only one type of hip joint pattern was sufficient: a duty-factor distorted sine-wave position signal (Figure 3, top plot). For lower speed gaits, double-peak knee signals (Figure 3, mid figure) were necessary to produce stable gait patterns. For higher speed, both single-peak (Figure 3, bottom plot) and double-peak signals produce stable gait patterns.

### 4.1. Impact of mechanical entrainment

For all experiments mounting the robot on a stand and moving legs *in-air*, gaits with double-peak knee signals (DP-trot and DP-walk) showed three principal components accounting for at least 95% of the variance. All experiments *on-ground*, independent from the robot speed, showed four basic patterns. These results support the first hypothesis (H1-1), derived from observations with animals; level-ground locomotion showed four to five basic components (Ivanenko et al., 2004; Dominici et al., 2011; Moro et al., 2013a,b).

Our *in-air* observations (hypothesis H2) coincide qualitatively with observations from human *in-air* stepping exhibiting simpler, harmonic leg kinematics (Ivanenko et al., 2002), essentially a lower observed kinematic complexity. However, we were unable to find quantitative descriptions of PCs for *in-air* stepping in animals with feed-forward-only motor control. Until otherwise reported, we consider the occurrence of either 2 or 3 PCs for the *in-air* patterns corresponding to 4 *on-ground* PCs as a weak indication for a similar mechanism in animals. Only similarly conducted animal experiments could potentially reveal evidential details on feed-forward motor control mechanisms in animals.

The interaction of the robot with its environment (i.e., ground contact) increased the kinematic complexity by at least one principal component. Qualitatively, this was externally observable through emerging robot body pitch and roll patterns.

For the single-peak knee actuation experiment (SP, 3.5 Hz, trot) Cheetah-cub robot ran at high speed *on-ground*, in average 0.8ms^−1^. Replaying the same CPG drive signals *in-air* showed two basic patterns (98%, Figure 8C). We found stable *on-ground* SP-gait patterns only at higher robot speeds, from 0.55 to 1.1ms^−1^. From this speed on, the robot's leg springs were sufficiently deflected by inertial forces and gravity, acting on the robot body, and enabled mechanical entrainment. Four basic patterns accounted for at least 95% of the variance of *on-ground* locomotion, for the SP-gait experiment. This result is not in accordance with the second part of hypothesis 1 (H1-2); a decreasing number of *on-ground* PCs was found at higher animal speeds for cockroaches (Koditschek et al., 2004). However, Cheetah-cub robot is unable to perform normalized speeds documented for these insects. The maximum robot speed recorded was 6.9 body lengths per second, or (Froude number of FR = 1.3 Spröwitz et al., 2013). It is possible that Cheetah-cub is not running fast enough to replicate similar results. Above results have potential implications for the general implementation of quadruped robot locomotion controllers: at level-ground running, the resulting patterns *on-ground* require not more than four basic components. This reduces the necessary complexity of the locomotion controller, also for other controller types than CPGs.

### 4.2. Control dimension reduction and robotic gait generation

The CPG model used for the control of locomotion included more that 10 open parameters, tuned for each gait. We hypothesize that with a collected dataset of sufficient size, the extracted in-air PCs representing this dataset can be used to reconstruct a new controller. Hence, to generate a new gait, one could tune the in-air primitive-weights for different joints, instead of CPG control parameters. However, many additional experiments are required to prove this claim. If above weight-tuning would apply, one could also encode the extracted primitives into a Dynamical Movement Primitive (Ijspeert et al., 2003, 2013). This would provide smooth modulation of the output signals as well as feedback incorporating capabilities (Ajallooeian et al., 2013). Such a controller would allow for switching between different gaits, by a change of primitive weights.

At present, we found no quantitative data available to compare our results with other legged robotic platforms, to observe the effect of mechanical entrainment of feed-forward controlled, legged robots and their kinematic motion primitives. Platforms other than Cheetah-cub robot exist, locomoting with dynamical, feed-forward controlled, self-stabilizing gaits and similar leg mechanics. Bobcat-robot for example is a small bounding robot and it can reach dynamical, full flight phases in-between touch-downs. It is equipped with a Cheetah-cub-like feed-forward controller, in-series elastic segmented legs, and an actuated spine (Khoramshahi et al., 2013). However, no data on the complexity of its kinematic *on-ground* PCs is available. Typically, motor controller designs for legged robots feature explicit feedback loops (Kimura et al., 2007; Buchli et al., 2009).

Mechanical compliance in legged robotic design has been introduced very early; Raibert's robots featured in-series elasticity (air springs, Raibert et al., 1984). Raibert reported a closed-loop controller with explicit, though simple and linear feedback. We are unaware on how much Raibert's machines could have been controlled in a feed-forward manner. We experienced that sufficiently complex feed-forward CPG signals (two to three PCs, speed dependent), a segmented leg design, and in-series and in-parallel leg compliance were the necessary ingredients for a simple, yet self-stable, dynamically legged quadruped robot system. Cheetah-cub and other robots (Iida and Pfeifer, 2004; Khoramshahi et al., 2013) are indicators that a larger design pool for mechanically entrained, dynamical robots exists.

As for the robot's range of leg compliance; Cheetah-cub features a slightly lower leg stiffness than observed in animals of the same weight and leg length. It would be an interesting future experiment to incrementally alter the leg stiffness, up to a level where the leg has no compliance. This would also require a way to alter the feed-forward control patterns in a systematic way, to ensure comparability between resulting gaits.

### 4.3. Relevance to biological systems

Cheetah-cub is a bio-inspired robot designed and motion controlled according to bio-inspired blueprints. Therefore, it presents a strong abstraction. We replicated observed blueprints from functional anatomy (pantograph leg, in-series and in-parallel compliance, clutch mechanism) and control (CPGs and locomotion parameters: duty factor, leg length, leg angle, amplitude, and frequency). The applied feed-forward controller produced position-control motor signals. Swing-leg dynamics are different to that of an animal, because its mechanical spring force cannot be manipulated in an online fashion. The robot's distal compliance acts passively and in-series, whereas quadruped animals are able to adjust ankle stiffness. Cheetah-cub does not feature an antagonist actuator producing a foot-joint stiffness profile.

Studies on legged locomotion in biology indicate that 4–5 principal components account for a large part of kinematic data variance, both for vertebrates and invertebrates (Ivanenko et al., 2004; Holmes et al., 2006; Dominici et al., 2011; Moro et al., 2013a,b). This is remarkable, considering the range of leg lengths, body sizes and weights, and differences in leg design and actuation strategies. In our study, four PCs accounted for 95% of the variance of all level-ground experimental data, including walk and trot gait data. The corresponding locomotion controller (CPG in feed-forward mode) was programmed with changing complexity. Depending on the range of robot speed either 3 or 2 PCs accounted for the variance of the feed-forward controller data (slower and faster *in-air* patterns, respectively). Perturbation experiments with running birds (guinea fowl) indicate that bipedal running is controlled through a combination of feed-forward control and additional, reflex-based actuator changes (cf. Daley et al., 2006, 2009). For future robotic legged experiments it will become interesting to observe and quantify the effect of explicit, possibly reflex-based feedback. Adult human locomotion patterns showed more pronounced basic gait patterns, compared to those of toddlers (cf. Dominici et al., 2011). These patterns were also phase-locked at important gait events. It is also unclear if and how a faster running robotic system would reduce the number of observable principal components with increasing robot speed, similar to the findings of Koditschek et al. (2004).

## 5. Conclusion

In this study we reported on the interplay between a modular, feed-forward locomotion controller, and the mechanical entrainment of a quadruped, self-stably walking and trotting compliant legged robot. We measured the complexity of the feed-forward controller, and the complexity of the resulting leg kinematics through the number of basic patterns accounting for a certain variance of kinematic data from *in-air* leg motions and *on-ground* locomotion, respectively. We implemented lateral sequence walk and trot gaits, and applied two different leg length control strategies. We found that the number of basic patterns from *on-ground* locomotion data matched those reported for animals; four basic patterns accounted for ≥95% of the variance. Three basic patterns accounted for ≥95% of the variance in lateral sequence walk and slower trot *in-air* experimental kinematic data, and two basic patterns accounted for faster trotting. Because patterns were sent in a feed-forward manner, the measured complexity of *in-air* kinematic data represents the complexity of the feed-forward controller. This shows that already a simple, modular rhythm generator is sufficient for level-ground, feed-forward legged quadruped locomotion, for two different gaits walk and trot. It also shows that passive mechanical compliance enables an increase of kinematic complexity, leading to dynamic and self-stabilizing walk and trot locomotion. In the case of our quadruped legged robot, the complexity of the kinematic data increased at ground contact, through mechanical entrainment between the feed-forward controller and compliant, bio-inspired robot hardware. Here, the bio-inspired leg design supported the emergence of additional *on-ground* basic primitives, e.g., through passive leg compliance and leg segmentation. Animals show a much wider range of tools to adapt and modulate dynamic legged locomotion. Similar results between presented robot experiments and experiments with animals at level-ground locomotion indicate that modular, feed-forward, rhythmic pattern-based motor control in combination with compliant hardware are important components of animal neuro-control and bio-mechanics.

### Conflict of interest statement

The authors declare that the research was conducted in the absence of any commercial or financial relationships that could be construed as a potential conflict of interest.
